# Aromatase inhibitors in advanced breast cancer: there are efficacy differences

**DOI:** 10.1038/sj.bjc.6602434

**Published:** 2005-03-15

**Authors:** A S Bhatnagar

**Affiliations:** 1 World Wide Services Group Ltd, Geispelgasse 13, Muttenz CH-4132, Switzerland

**Sir**,

In the recently published review, ‘Aromatase inhibition in the treatment of advanced breast cancer: is there a relationship between potency and clinical efficacy?’ (Sainsbury, 2004), the author claims that differences in the potency of aromatase inhibition and oestrogen suppression among the modern aromatase inhibitors do not translate into differences in efficacy benefits.

The clinical trial results listed within the article, however, contradict the author's conclusion. Furthermore, some of the more general statements within this review must be challenged.

Dr Sainsbury claims to see no *efficacy differences between letrozole and anastrozole*, despite the former being associated with higher potency in aromatase inhibition and oestrogen suppression (Geisler *et al*, 2002). The author makes a number of indirect comparisons of different trials to support his point: the first-line advanced breast cancer trials of anastrozole or letrozole *vs* tamoxifen demonstrated ‘anastrozole to be at least as effective as tamoxifen’ (Bonneterre *et al*, 2001; Nabholtz *et al*, 2003), whereas ‘letrozole was found to be superior to tamoxifen’ in all end points including a prospectively planned survival analysis at 1- and 2-year follow-up, which is not mentioned in this review (Mouridsen *et al*, 2003).

In a head-to-head comparison of anastrozole *vs* letrozole in second-line advanced breast cancer, results were comparable in all end points except overall response rate (ORR), which was more than 50% higher in patients treated with letrozole (Rose *et al*, 2003). While ORR was a secondary rather than primary end point of the trial, the higher likelihood of responding to endocrine therapy is inarguably highly relevant for women with advanced stage breast cancer, as response implies both personal relief as well as delay of more aggressive, usually cytotoxic, therapies.

Dr Sainsbury uses a subgroup analysis to challenge the results of this 713-patient trial, citing a significant superiority for letrozole only in the group of patients with undetermined hormone receptor status. Letrozole response rates in this group of patients were similar to those in the overall trial collective, not surprisingly so, as at least two-thirds of these patients are likely to bear oestrogen receptor (ER)- and/or progesterone receptor (PgR)-positive tumours. In view of the size of the ER/PgR-unknown subgroup (*n*=340), the well-balanced demographics in the two trial arms overall, and the high chance of a tumour of undetermined hormone receptor status being positive renders statistical imbalance of ER/PgR status in favour of letrozole, a very unlikely explanation for the 2.5-fold increased response rate achieved with letrozole.

In fact, the high efficacy of letrozole in ER/PgR-unknown tumours in this second-line study is consistent with results of the phase III first-line comparison of letrozole *vs* tamoxifen, in which the superiority of letrozole over tamoxifen was consistent among both ER/PgR-positive and -unknown patients – in contrast to the anastrozole trials that demonstrated superiority only in the ER/PgR-positive subgroup. While hard to explain, it seems that while letrozole may be effectively used in both hormone receptor-positive and -unknown patients, the efficacy of anastrozole appears limited to only those patients with a distinct expression of ER/PgR.

The *efficacy of an endocrine agent in ER/PgR-unknown patients* remains clinically relevant, as even today there is a pool of patients with advanced breast cancer of undetermined ER/PgR status. While Dr Sainsbury maintains that ‘…they [AIs] are used in this [hormone receptor-positive] group of patients’, this is, in fact, not entirely true for advanced breast cancer: endocrine therapy is still widely recommended for these women with receptor-unknown advanced breast cancer, based on the fact that the majority will be endocrine responsive and a response to endocrine therapy would delay chemotherapy (National Cancer Institute, 2004).

Within the summary of this review, the author introduces the notion that while the *degree of oestrogen suppression* beyond a certain threshold does not translate into increased antitumour efficacy, it might well result in different toxicity profiles. This statement is purely speculative and is not supported by any data available so far. We are not aware of any research demonstrating that endocrine-sensitive normal tissues, such as bone, would be more sensitive to small changes in oestrogen levels than breast cancer cells, which are well recognised for their ability to develop a pronounced hypersensitivity to small levels of oestrogen (Masamura *et al*, 1995). Using bone metabolism as an example, we may point out that the less potent aromatase inhibitor anastrozole has been associated with a marked increase in fracture rates when used in early breast cancer (Baum *et al*, 2002, 2003), whereas letrozole has not (Goss *et al*, 2003).

The author of this letter is a consultant to Novartis Oncology. It would seem advisable that future reviews of the kind published by Dr Sainsbury include disclosure of the author's potential conflicts of interest as well.

## References

[bib1] Baum M, Buzdar AU, Cuzick J, Forbes J, Houghton JH, Howell A, Sahmoud T, for the ATAC (Arimidex, Tamoxifen Alone or in Combination) Trialists' Group (2003) Anastrozole alone or in combination with tamoxifen versus tamoxifen alone for adjuvant treatment of postmenopausal women with early-stage breast cancer. Results of the ATAC (Arimidex, Tamoxifen Alone or in Combination) trial efficacy and safety update analyses. Cancer 98: 1802–1810 14584060 10.1002/cncr.11745

[bib2] Baum M, Buzdar AU, Cuzick J, Forbes J, Houghton JH, Klijn JG, Sahmoud T, for the ATAC (Arimidex, Tamoxifen Alone or in Combination) Trialists' Group (2002) Anastrozole alone or in combination with tamoxifen versus tamoxifen alone for adjuvant treatment of postmenopausal women with early breast cancer: first results of the ATAC randomised trial. Lancet 359: 2131–2139 12090977 10.1016/s0140-6736(02)09088-8

[bib3] Bonneterre J, Buzdar A, Nabholtz JM, Robertson JF, Thürlimann B, von Euler M, Sahmoud T, Webster A, Steinberg M, for the Arimidex Writing Committee; Investigators Committee Members (2001) Anastrozole is superior to tamoxifen as first-line therapy in hormone receptor positive advanced breast carcinoma. Cancer 92: 2247–2258 11745278 10.1002/1097-0142(20011101)92:9<2247::aid-cncr1570>3.0.co;2-y

[bib4] Geisler J, Dowsett M, Lønning PE (2002) Letrozole is more effective than anastrozole in suppressing total body aromatization and plasma estrogen levels in postmenopausal breast cancer patients. Am J Oncol Rev 1: 99–102 10.1200/JCO.2002.20.3.751 11821457

[bib5] Goss PE, Ingle JN, Martino S, Robert NJ, Muss HB, Piccart MJ, Castiglione M, Tu D, Shepherd LE, Pritchard KI, Livingston RB, Davidson NE, Norton L, Perez EA, Abrams JS, Therasse P, Palmer MJ, Pater JL (2003) A randomized trial of letrozole in postmenopausal women after five years of tamoxifen therapy for early-stage breast cancer. N Engl J Med 349: 1793–1802 14551341 10.1056/NEJMoa032312

[bib6] Masamura S, Santner SJ, Heitjan DF, Santen RJ (1995) Estrogen deprivation causes estradiol hypersensitivity in human breast cancer cells. J Clin Endocrinol Metab 80: 2918–2925 7559875 10.1210/jcem.80.10.7559875

[bib7] Mouridsen H, Gershanovich M, Sun Y, Perez-Carrion R, Boni C, Monnier A, Apffelstaedt J, Smith R, Sleeboom HP, Jaenicke F, Pluzanska A, Dank M, Becquart D, Bapsy PP, Salminen E, Snyder R, Chaudri-Ross H, Lang R, Wyld R, Bhatnagar A (2003) Phase III study of letrozole versus tamoxifen as first-line therapy of advanced breast cancer in postmenopausal women: analysis of survival and update of efficacy from the International Letrozole Breast Cancer Group. J Clin Oncol 21: 2101–2109 12775735 10.1200/JCO.2003.04.194

[bib8] Nabholtz JM, Bonneterre J, Buzdar A, Robertson JF, Thürlimann B (2003) Anastrozole (Arimidex™) versus tamoxifen as first-line therapy for advanced breast cancer in postmenopausal women: survival analysis and updated safety results. Eur J Cancer 39: 1684–1689 12888362 10.1016/s0959-8049(03)00326-5

[bib9] National Cancer Institute (2004) Breast cancer (PDQ®): treatment. Available at: http://www.nci.nih.gov/cancertopics/pdq/treatment/breast/healthprofessional (accessed August 20, 2004)

[bib10] Rose C, Vtoraya O, Pluzanska A, Davidson N, Gershanovich M, Thomas R, Johnson S, Caicedo JJ, Gervasio H, Manikhas G, Ben Ayed F, Burdette-Radoux S, Chaudri-Ross HA, Lang R (2003) An open randomised trial of second-line endocrine therapy in advanced breast cancer: comparison of the aromatase inhibitors letrozole and anastrozole. Eur J Cancer 39: 2318–2327 14556923 10.1016/s0959-8049(03)00630-0

[bib11] Sainsbury R (2004) Aromatase inhibition in the treatment of advanced breast cancer: is there a relationship between potency and clinical efficacy? Br J Cancer 90: 1733–1739 15150604 10.1038/sj.bjc.6601731 PMC2410276

